# Extra Supplement—The Nursing Mirror

**Published:** 1889-08-03

**Authors:** 


### The Nursing Mirror.

All Contributions for this Supplement should be addressed to the Editor, The Hospital, 139-140, Salisbury Court, Fleet Street, London, E.C., and should have the word "Nursing" plainly written in left-hand top corner of the envelope.

### En Passant.

NURSE-STEWARDESSES.—The register of nurse-stewardesses has been printed and circulated amongst shipping companies. Any nurse desiring to see the register can do so by applying at the office of The Hospital, 139, Salisbury-court, any day of the week except Saturdays.

THE DANGERS OF A REGISTER.—A letter appeared in last week's *Lancet* from Mr. Russell Combe, F.R.C.S., which we should have liked to reproduce had we the space. Mr. Russell Combe saw some of the dangers of a register very clearly, but did not seem to quite understand how it would debase all nurses to one dead level. One fact mentioned in the letter was that the forty nurses of the Devon and Exeter Hospital, without exception, refused to join the British Nurses' Association, though Miss Wood visited them and lectured them on the subject. We think this fact is highly creditable to the keen foresight and intelligence of the Exeter nurses.

A WARNING TO NURSES.—Mr. Charles Lunn has been made a bankrupt, and the following facts came out at his examination. The bankrupt said that in March last he took a lease of 97, Gloucester-place, at a rental of £260 a year, and opened the premises as "The Institution for Trained Nurses." His only means at the time were £20 or £30 of borrowed money, but he had expectations from some distant relatives of his wife. He obtained the furniture for the institution on credit from Messrs. Collinson and Lock, Messrs. Marshall and Snelgrove, and other firms. Before Messrs. Collinson's goods were delivered he signed a document setting out that he had an income of from £500 to £800 a year. He must have meant that he expected the business he was about to start would produce that income. Anyone seems to have the right to start an institution and get nurses to make it pay for them. Mr. Lunn has failed, but we know some who have succeeded. Nurses cannot be too particular as to what institutions they join.

HARRIETT BROWNLOW BYRON.—On July 19th, the Duchess of Albany laid the foundation-stone of the Eastbourne Hospital for Children, to be erected as a memorial to the late Miss Byron, Mother Superior of All Saints, Margaret-street. The life of Miss Byron is worthy of commemoration; it was one long course of self-denial and gracious charity. Having gone through a course of training as nurse in King's College Hospital, Miss Byron, in 1851, opened a small home for incurables and orphans in Margaret-street, London. The fruit of that comparatively small work is still ripening; for the Sisters of the Community founded by Miss Byron now number over three hundred, and are working everywhere for the poor, their lives being absolutely dedicated to this purpose. In England these Sisters have twenty-six institutions, and they have branches in Scotland, America, Africa, and India—Orphanages, Maternity Homes, Training Schools, Convalescent Hospitals, Incurable Hospitals, Homes for Fallen Women, Training Schools for Nurses, etc., and they also have charge of the sick at University College Hospital. During the Franco-Prussian War Miss Byron organised a little band of Sisters and went out with them to the scene of battle, and during the campaign these heroic ladies attended the wounded soldiers and those laid low by cholera, typhus, and smallpox. One of Miss Byron's greatest achievements was founding the Convalescent Home at Eastbourne, which is now to have such an excellent addition. Miss Byron died in 1887, and another sister now wears the insignia of office in her stead.

ANOTHER PARISH NURSE.—The rector of Carshalton, Surrey, has profitted by the example shown at Sutton, and has started a district nurse to attend to the poor of his parish. The day when every district shall have its nurse as surely as it has its doctor is fast approching.

NORWICH NEWS.—The report of the Bethel-street Nurses' Home is out, and tells of 260 cases attended, representing 1,132 weeks of nursing. Forty-nine cases were nursed gratuitously, and 53 at reduced fees. The committee have closed the year clear of debt, but have a very small balance in hand. They regret they receive no help from the Sunday Fund. Two nurses have left the staff, and six have been added to it in the course of the year, the number of nurses now at work being 27. Seven probationers are in course of training—three at St. Mary's Hospital, Paddington, and four at the Infirmary, Derby.

A SUNDERLAND ECHO.—The Hartley Wing of Sunderland Infirmary is not to be nursed by the Tottenham community, who have charge of the main building. The reason is said to be that there are not enough deaconesses ready to enter for training, but Mr. Wilkinson hints that if the deaconesses were better paid they might be more numerous. A labourer is always worthy of his hire, and there is no reason why a deaconess should not be given the usual wage. What though she has forsaken the pomps and vanities of this wicked world, still the money would be useful to the community. Certainly all will agree it is a pity to have two different classes of nurses in one infirmary.

A NURSE'S SOPORIFIC.—An amusing sketch of a Sister of Charity is given in a novel lately published. Sister Brigitte carries in her pocket a little old copy of the "Lives of the Saints," and whenever she thinks her patient should sleep, she produces the book and commences to read wherever the book opens, and goes steadily on in a monotonous voice till the desired effect is produced. Sister Brigitte is a nurse of the old school, who confines her notion of duty to simple unfaltering obedience to the doctor. It so happens the doctor is not an exemplary character; he is jealous of the patient on whom Sister Brigitte waits, and disfigures him for life by giving him a long course of mercury. We should like to see a modern nurse, learned in all the ologies, sitting calmly by while a patient with rheumatic fever was turned blue by mercury! Obedience, apparently, has its faults.

THE RED CROSS IN HOLLAND.—Some account of this work was given at the Paris Congress. The Committee of the Red Cross at Hague give an annual class, presided over by an army doctor, students are giving instruction in the first aid to be given to the wounded, and the practice of dressing wounds and bandaging takes place three times a week under the direction of a surgeon and a sister-nurse. These classes are followed by the members of the committee and by women desirous of making this their profession. The latter are obliged to pass a period of about six months in the town hospital, after which they are examined and certificated, and are then in great demand as sick nurses. The committee, a short time age, hired a house, where the sisters who have diplomas find a home at moderate prices, while the apprentice nurses are lodged and boarded there at the expense of the committee. A large room on the ground floor and a bathroom adjoining are adapted for the antiseptic treatment of the wounded or infirm poor. The committee dispense very little money—300 florins for general expenses—but the zeal of its members knows no bounds, and this has allowed in the past year for the care of 170 wounded and 1,629 wounds being dressed.

### Physiology for Nurses.

Baths and Their Effects. *(Continued.)*

Those who avoid cold baths are the very young and the very old, because they cannot resist the shock of the sudden plunge. It is a great mistake to try and harden children, as it is called, by giving them a cold bath. They probably dislike it on account of the disagreeableness following. Moreover, in all probability it will give them a dread of cold water for the rest of their lives. The use of the hot bath is to give a more thorough cleansing to the skin than would be possible with cold water, since a hot bath is more agreeable on the whole, and a person can remain in it a longer time. The effect on the skin is to increase the amount of blood to the surface, and thus to relieve the internal organs by depriving them of blood. If indulged in too frequently and too hot, these baths have a weakening effect, and also make a person more susceptible to cold. A very hot bath should be used with great caution. Another use of a hot bath is to soothe after great fatigue, and often, when it is impossible to get a proper amount of sleep, a hot bath will enable a person to dispense with a few hours' sleep. After great activity, the muscles become wearied with the long strain put on them, the waste products collect, and, the rest of the body being overtired, it cannot act, and these waste products are not thrown off and fresh ones formed to take their place. But the hot water carries off the waste at the same time that it helps the body, by warming it, to bring fresh material to supply the waste; and this is what would have happened during sleep, only more slowly. Whenever it is possible, a hot bath should be taken before going to bed, because the effect caused by the hot water can be carried on in bed, and cold is avoided when the skin is most likely to feel it.

Soap, that most necessary addition to the bath, is made of various fats boiled down with caustic soda. The worst of it is that soaps are terribly adulterated; such things as British gum, starch, gelatine, pumice stone, etc., are used, though as only the gum dissolves in water, the others can be easily diluted, as they settle at the bottom as a sediment. Cocoa-nut oil makes a soap which will lather in sea-water, and is useful on long voyages for that reason. Soaps are coloured with various things: red soap is made so by vermilion, which is poisonous, and should be avoided. The common household yellow soap is often very wet, and when dried shrinks a great deal. This is due to the way in which it is made, by which it is made to weigh more, and does not prove that it is a good kind.

Turning now to the consideration of the hair and nails: even as long ago as the days of the old Greeks and Romans, the women wore their hair long and the men short; whilst the old inhabitants of France, both men and women, wore their hair long, but when conquered by Julius Cæsar, he made them cut their hair short as a sign that they were conquered. It was partly on this account, and partly because long hair was looked upon as a great ornament and women vied with one another in the thickness and length of their hair, that when anyone was about to become a monk he shaved his head, by which he showed he gave up all earthly adornments and vanities and submitted himself to his spiritual masters. Later on, in France the kings only, and those connected with the royal house, were allowed to wear their hair long, whilst every other man had to wear it short. The old inhabitants of England also wore their hair long, both men and women, and prided themselves very much on its length and beauty. It is said that a young soldier who was condemned to be beheaded, begged that no slave should touch his beautiful long hair nor that it should be stained with blood. Even in those days those old inhabitants were not satisfied with the natural colour of their hair, but tried to improve it with various simple dyes. Later on, young women before marriage were allowed to wear their hair long and uncovered, and flowing over their shoulders, but on marriage it had to be cut shorter and tied up, and they had to wear a head-dress of some kind. Soon the clergy were much annoyed that they should be distinguished from the laity by their short hair, so they began to preach against long hair, and Anselm, a certain Archbishop of Canterbury, preached before the king against the wickedness of wearing long and curled hair; and his sermon had such an effect upon the king that he decided to have his long hair cut off. The archbishop was prepared for this result and produced a large pair of shears, which he had hidden up his sleeve, and cut off the hair of the king and his courtiers. Of course, for a time it was the fashion to have short hair, but it did not go on for long, and they returned to their flowing locks once more, until, so goes the story, a certain knight dreamt that he was suffocated by his hair, and on waking he cut it off. This absurd report is said to have spread all over England and everyone cut their hair off. This lasted for a year, and then, says the writer, "all who wished to appear fashionable returned to their former wickedness and contended with the ladies in length of hair." Thus, sometimes long hair and sometimes short was the fashion until we come to the period when those huge wigs and powder were used. This powder was largely made of starch, and to obtain enough starch a great deal of corn had to be used, and so in times when bread was scarce, one of our great statesmen put a tax on corn, and hence it was no longer used to get the starch from it out of which to make the hair powder. Now, of course, everyone follows their own taste and inclination, and wear hair short or long as they find most convenient.

In thinking of the structure of hair it must not be imagined that it is anything peculiar to itself, it is merely part of the cuticle or outer layer of the skin, specially developed to be hair. The bulb of the hair, which can be seen when you pull a hair "up by the root," is, as it were, planted in the skin, and is nourished by a network of bloodvessels which run along each side of the hair and surround it. This part of the hair is, of course, below the surface of the skin of the scalp, and, therefore, is not seen. Above the skin the hair which we see is a very fine tube, the walls of which are formed of cells, and is very much like a lengthened out tube of skin, sufficiently hard to keep its shape. These cells are kept together by a kind of cement in which are embedded pigment cells, and the colour of the hair, like that of the skin, depends upon the amount of this pigment—fair hair has less pigment, the darker the hair the more pigment there is in these cells. However, in this cement there are also found air-bubbles, and when the hair becomes grey it is due to two causes: first, the disappearance of these pigment cells; and second, to the greater number of these air-cells, which give a sort of grey colour. The hairs, moreover, do not come straight out of the skin, but in a sloping direction, and therefore they lie flat on the head; if they came out straight they would stand up. Muscles are attached to each hair, which move it and make it very elastic. The hair can be curled because the heat of the curling-iron dries up the moisture on one side of the hair and make it contract or shrink, and as this shrinking is on one side only, it makes the hair bend or wave, just as when you hold a piece of paper to the fire it curves. Then, when the hair is weaker on one side than another it curls naturally, as it is called—*i.e.*, it bends towards the weakest side. All so-called curling fluids are made of some kind of oil which prevents the damp, which, as you know, makes the hair go straight again; as when you go out in damp weather, if you have curled your hair, it goes quite straight; in the same way the oil in curling fluids keeps the damp out of the hair, and the other part dries it, and so it curls. The use of the hair is to supply a covering for the head as a protection from heat and cold, whilst the eyelashes protect the eyes from dust by acting as a barrier to its entrance. As a rule, there are 600 hairs to every square inch of the head in those who have dark hair, and about 700 in those whose hair is light coloured, because the fairer the hair, the finer each single hair is; but many things make the hair coarse—such as exposure to the weather. Those who never wear hats have coarser hair than those who always keep the head covered. The strength of the hair, considering its thickness, is very great, each single hair, it has been found, will bear a weight of 4ozs. without breaking, and, as you remember, the weight that all the hair will bear is very great, since in Absalom's case he was hung up by it and it bore all the weight of his body without breaking.

*(To be continued.)*

### Nursing at Northampton.

The Northampton Institution for supplying Trained Nurses is one which deserves a larger degree of public favour and interest than has hitherto fallen to its share. The value of a hospital-trained nurse as an attendant in any serious case of accident or sudden emergency is plain. For skilful handling of a wound is needed the thoughtfulness of an expert, comprehending at a glance any special points demanding care; and the ability to receive technical instruction from a doctor who can confidently leave the patient till his next visit without fear that injudicious friends may in the interval render his work ineffectual—these things are now fairly appreciated and understood. But where the case is one rather of prevention than cure, the science of hygiene, or things which tend to health, is still very insufficiently known to those who have not made it a special subject of study. Consequently the value of hospital training in the "art of sick nursing" is apt to be disregarded. Care of an ordinary sick person is considered to be within the range of most untutored faculties: cleanliness, cheerful surroundings, fresh air, simple and suitable food, quietness as a means of healing. Such things are still frequently scorned, as worthy of attention only from the fastidious and the fanciful. It is an enterprise worthy of English civilisation to combat the dogged indifference to healthy life, and the remorseless disregard of entailed suffering that exists on every hand in our crowded cities and villages.

Lectures are given, articles in magazines are written and published, ambulance classes are held, much is already done to inform the public mind on these subjects, but certainly one of the most obvious methods, the most simple and the most sure, is the presence of the trained nurse herself in the home of the sick person. The confidence she will inspire during a mere half-hour's visit will give assurance that her suggestions are reasonable and useful. In future her arrival will be looked for as sure to be productive of fresh arrangements for the relief and comfort of the sufferer, and in many a poor district her advice will be passed on to admiring neighbours, who may learn more common-sense which they will make practical use of in this simple fashion than they would ever get at through books or lectures. Northampton is happy in the possession of an institution which during the past twelve years has done good service to the cause. Its centre of work is at the Nurses' Home, 35, Hazlewood-road. A lady has charge of it as the superintendent, with a staff of ten of twelve hospital-trained nurses, who are in constant attendance on the sick, and return to the institution as their headquarters in the intervals of work. Let it be understood that to maintain a nurse in good health and fit for work she must be well fed, sufficiently clothed, and comfortably housed; she requires proper rest, and plenty of fresh air. To set it at a low average, a nurse's maintenance will cost £40 to £50 a year. If she is required to spend her time in attendance on the poor, for which no payment is asked or given, the payment must come from the charity of the rich. House rent and taxes for the home have also to be provided. Thus it is clear that an institution such as we speak of, without endowment, must depend to a certain extent upon charity. And this was bestowed upon the Northamptonshire Nurses' Home to the extent of £457 19s. in donations and subscriptions in the year 1878 as shown by its first annual report; but this voluntary aid has in the succeeding ten years not exceeded a quarter of the sum above mentioned, and although an excellent and almost supporting work has been done by some ten of the trained nurses' attendance on paying patients, the difficulty has been very great in maintaining the charitable part of the nursing work for which the institution was at first planted at Northampton. The benefit of the charity to the very poor in this town of 70,000 inhabitants is beyond description. Two "district nurses" are employed and maintained by the voluntary funds before mentioned, together with a supply of such things as air-pillows, blankets, sheets, and other invaluable comforts to invalids, which are kept at the institution for temporary loan on the payment of a nominal weekly sum, the repair and supply of which, in consequence of their constant requisition and use, is a considerable item of expense. The visits paid by the two district nurses in 1887 were 6,181, and in 1888 were 6,907, and these are entirely gratuitous, the salaries of the district nurses being provided by the voluntary funds of the institution. There is immense scope for the extension of this valuable work to the populous towns and villages in the neighbourhood, and demands for nurses have constantly to be refused, owing to the small number that can be housed in the institution, which is miserably small and ill-suited to its good purpose; but there are no funds wherewith to provide a better house for the nurses' home. Any contributions will be thankfully received by the Lady Superintendent, 35, Hazelwood-road, Northampton, or may be paid to the treasurer of the Nursing Institution Fund at the Northamptonshire Union Bank.

### Everybody's Opinion.

*[Correspondence on all subjects is invited, but we cannot in any way be responsible for the opinions expressed by our correspondents. No communications can be entertained if the name and address of the correspondent is not given, or unless one side of the paper only be written on.]*

### THE PENSION FUND.

A Provincial Nurse writes: I wish that I could express my true feeling with regard to the remarkable generosity of those London merchants towards all nurses of this nation. Allow me, through The Hospital, to express to them and to you, as well as I can, how extremely thankful I am to you all for your disinterested and kind-hearted liberality, and to you, Mr. Editor, for the great amount of trouble you must have been at in bringing The Nurses' National Pension Fund to its present state. Many thanks, and unceasing thanks, are due to you all who have brought it through, in spite of so many difficulties and so much opposition; but opposition may have done good in bringing it more before nurses and the public; if so, then I must thank the opposers too. It is to be hoped that the portraits of our benefactors will appear in The Hospital. I wish a meeting could be held in London to propose a public vote of thanks to the promoters and workers of the scheme. I would travel to London on purpose to be present, if it were possible, though living at a considerable distance from town.

### REGISTRATION.

Courage writes: I don't know what "Sister Katherine" is hesitating for; we, here, are only country nurses, but directly we heard about the half-crown register we withdrew promptly from the B.N.A. We had no desire to go on that register in which matrons, midwives, nurses, and women connected with nursing for three years were being enrolled. Why, we expect next to hear that ward-maids and scrubbers are on this register, since the chief recommendation seems to be the possession of half-a-crown. What "Sister Katherine" has to do is to write immediately to the secretary to remove her name from the books, and on no account to permit it to appear on the register. I am sure this is the step many nurses must now be taking.

### REGISTRATION.

Don writes: May I suggest to "Sister Katherine" that she should read The Hospital regularly, and then even the collapse of the B.N.A. will not surprise her. For my own part, as a member, I wish it every success, but faithfully read all The Hospital says on the other side. I was pleased to notice, in spite of some dissent in so many big hospitals, there were a great many eminent men belonging to them whose names did not appear in the protest.

["Don" has overlooked the fact that the memorial was confined to teachers, examiners, managers, and matrons of the Nurse Training Schools. "The many eminent men" "Don" refers to, being neither teachers nor examiners, could not be asked to sign the memorial, as they were ineligible in consequence.—Ed.]

### Notices.

The Editor of The Hospital has much pleasure in offering to nurses three prizes of £5, £3, and £2 respectively for the three best dressed sets of dolls representing the nursing staff of any hospital. The dolls must not be shorter than four inches nor taller than twelve inches, and must represent the sister, charge-nurse, and probationer of the hospital. Any nurse intending to compete must send her name and address on a post-card, headed "Doll Competition," to the Editor of the Hospital, The Lodge, Porchester-square, London, W., before September 1. Further particulars will be announced later on, and arrangements will be made for a public exhibition of the dolls where all nurses will be able to see the show.

The Directress of the Hollond Institution, Nice, is much obliged to all the nurses who have answered her advertisements, but she only finds it possible to correspond with those most suited to her requirements.

Would any lady or gentleman be kind enough to send to a fever hospital old books, novels, or numbers of any kind? Parcels would be gratefully received by "Nurse," Infectious Hospital, Greasly, Cheshire.

### The Nurse's Bookshelf.

### A BOOK FOR THE POCKET.

We heartily welcome the third edition of Mr. Walter Pye's "Elementary Bandaging and Surgical Dressing," (Trübner). This is a book which is within the reach of all nurses, and should be in the possession of all. The price is only two shillings, and though clear and complete information is given on surgical subjects, the book will slip easily into an apron pocket. The first section deals with bandages, strapping and splints; the second with the dressings of wounds and burns, and the making of poultices and other applications; the third section deals with first aid to cases of accident or emergency. The illustrations are very numerous and very clear, especially those dealing with splints. The last chapter on case taking will prove very useful to those nurses in cottage hospitals and country districts who have to combine a dresser's duties with their own.

### Appointments.

[It is requested that successful candidates will send a copy of their applications and testimonials, with date of election, to The Editor, The Lodge, Porchester-square, W.]

North of England Children's Sanatorium, Southport.—Miss C. C. Smith has been appointed matron to this Sanatorium. She received her training at The Royal Hospital for Sick Children, Edinburgh.

Newark Hospital.—Miss Maud Calvert, whose appointed as Sister at St. Saviour's Infirmary we chronicled 16 months ago, has been appointed matron at Newark. Miss Maud Calvert trained at Guy's.

Guy's Hospital.—Miss Florence Nott-Bower is leaving Huddersfield and going to return to her old friends at Guy's, where she is to assist the matron especially in superintending the nurses. In June, 1888, we noted Miss Nott-Bower's appointment to Huddersfield; we like to follow the careers of our friends in the nursing world.

### Presentation.

Mr. Christopher Bulteel has been presented with an ebony and silver paper-knife by the nursing staff of the Royal Devonport Hospital. Mr. Bulteel is retiring from practice.

### Vacancies.

The following vacancies are announced:

### Matrons.

Birmingham Workhouse (assistant), £40.
Kensington Infirmary, £100.

### Nurses.

Barrow-in-Furness Hospital (night), £20.
Birmingham Fever Hospital (several assistant).
Birmingham Orthopœdic Hospital.
Bow-road Infirmary, £18.
Brighton Borough Hospital, £28.
Cheltenham Institution.
City Hospital, Liverpool, £25.
Civil Hospital, Gibraltar, £25.
Eastern Fever Hospital, £22.
Glasgow Nursing Association (several), £25.
Hampstead Home Hospital, £22.
Holborn Union (night), £20.
Kendal Hospital (night), £28.
Kent Nursing Institution.
Liverpool City Hospital (two assistant), £25.
Liverpool Stanley Hospital, surgical
Maidstone Hospital, £26.
Manchester Consumptive Hospital, £20.
Morley's Convalescent Home (night), £16.
National Hospital, Queen-square.
Northwood Fever Hospital, £28.
Nurses' Home, Bengeo, £30.
3, Queen's-terrace, Glasgow.
Rotherham Hospital.
39, Royal-avenue, Chelsea.
Southampton Institute.
Stephen Monckton Nurses' Home.
Stepney Hospital for Children, £25.
Stratford-on-Avon Hospital, £25.
St. Saviour's Infirmary, Dulwich (several), £20.
West London Hospital (assistant), £24.

### District Nurses.

Moore, Cheshire.
1, Regent-street, Nottingham.
Queen Victoria Institute, Edinburgh.

### Probationers.

Barnet Cottage Hospital.
Barrow-in-Furness Hospital, £16.
Batley Cottage Hospital.
Coldstream Cottage Hospital.
Manchester Consumptive Hospital.

### Notes and Queries.

To Correspondents.—1. Questions may be written on post-cards. 2. Advertisements in disguise are inadmissible. 3. In answering a query please quote the number. 4. If a private answer is desired, a stamped addressed envelope must be forwarded.

### Queries.

(55) *Massage.*—Could you tell where massage is taught, and whether it is better to learn in a hospital or privately? *M. B.*

(56) *Deaconesses.*—Can you give me particulars of the work and pay of deaconesses? *Monica.*

(57) *Nursing Lectures.*—Where could I see something of hospital nursing without actually becoming a paid nurse? *Ada.*

(58) *Asylum Nursing.—Inquirer* asks: Could any nurse kindly say the best asylum in England where a respectable woman could be thoroughly trained as medical and mental nurse? What books are necessary to study on insanity?

(59) *A. H.* wishes to know if there are any cottage hospitals or similar institutions where a lady, who has been six months in a London hospital, could go for a few months for the purpose of gaining more knowledge in sick nursing.

(60) *Nursing in New Zealand.—R.* writes: Is there any English agent where information can be had and particulars of nursing institutions in New Zealand?

### Answers.

(51) *Trained Nurse* should watch the daily papers. A lady advertised in the *Telegraph* about three weeks ago for someone to accompany her to Australia to assist with child in return for passage. I am sorry I cannot give the address. *Doris.*

(51) *Trained Nurse* should write to the Secretary of the Orient Line, 25, Cockspur-street, Charing Cross, and explain her case and enclose copies of her testimonials. They might be able to tell her of some passenger needing a nurse or a maid for the voyage.

(52) *J. F. H. M.* should apply to Mrs. Blackwell, Highlands, Menchin Hampton; or Mrs. Dalton, Little Burstead Rectory, Brentwood; or to Mrs. Ernald Smith, The Oaks, Emsworth.

(53) *A. J. R.*—The waterproofs or cloaks are made and kept at the infectious hospitals, and every visitor is lent one for the time being.

(55) *Massage.*—Apply to the West-end Hospital, 73, Welbeck-street, or the Lady Superintendent, 317, Clapham-rise. If you prefer private teaching, apply to Mrs. Creighton Hale.

(56) *Deaconesses.*—"Monica" should address herself to the Head Deaconess, 2, Sutton-place, Hackney, E., for full particulars. The Bishop of Bedford is president of the home. "Monica" would be at liberty to enter the home as a visitor, in the first instance, at a charge of a guinea per week. If she found that she had a distinct aptitude for Church work, she could then become a probationer, paying £50 a year. At the end of this period of training, if considered suitable, she would be presented to the bishop, who would set her apart for the deaconess office. Deaconesses are of two kinds—sister deaconesses or unattached. In the latter case they are free to take work in any district they please, provided the bishop's sanction is previously obtained.

(57) *Nursing Lectures.*—Apply to the Home Hospital for Ladies, Lupus-street; non-residents are admitted to classes and lectures there.

*Nurse.*—The St. John Ambulance Handbook of First Aid, price 1s., is what you want. It can be had from the local secretary for the St. John Ambulance Association, or from the secretary, St. John's Gate, Clerkenwell, E.C.

